# Reaching high-hanging fruit in antimicrobial stewardship: a hospital-based intervention to withdraw inappropriately prescribed antimicrobials

**DOI:** 10.1017/ash.2024.48

**Published:** 2024-04-16

**Authors:** Salman S. Khan, Ilya Krichavets, Marta Feldmesser

**Affiliations:** 1 Zucker School of Medicine at Hofstra/Northwell, Hempstead, NY, USA; 2 Department of Pharmacy, Lenox Hill Hospital, New York, NY, USA; 3 Division of Infectious Diseases, Lenox Hill Hospital, New York, NY, USA

## Abstract

**Background:**

Antimicrobial stewardship programs (ASPs) are responsible for addressing unnecessary antimicrobial use. We describe our experience with a unique intervention to withdraw unnecessary antimicrobials.

**Methods:**

Design, Setting, Participants: descriptive case series of adult inpatients at a single academic medical center, December 2021 to December 2022; Intervention: hospital-wide policy allowing ASP to discontinue inappropriate antimicrobials in select cases not resolved by prospective audit and feedback; Measures: count, date, and generic names of antimicrobials prescribed; reason for antimicrobial withdrawal (prolonged duration, no evidence of infection, or other); withdrawals by inpatient service (surgical or medical); time from antimicrobial start date to withdrawal intervention; days of therapy (DOT) saved; “nudge effect” defined as the prescribing team self-discontinuing withdrawn antimicrobial within 24 hours of withdrawal notice; appeals to withdrawals; ordering of alternative antimicrobials following withdrawal; incident infections, readmission, in-hospital mortality within 30 days of withdrawal intervention.

**Results:**

There were 54 antimicrobials withdrawn among 36 unique patients during the study period; piperacillin-tazobactam followed by vancomycin were the most frequently withdrawn agents; prolonged duration of therapy or prophylaxis followed by no evidence of infection were the most common reasons for withdrawal; withdrawals occurred most often on surgical services; an estimated 236 DOT (27.2 DOT per 100 patient-days) were saved; 32% of withdrawals were appealed; alternative antimicrobials were ordered following 20% of withdrawals; no incident infections, readmissions or in-hospital deaths were definitively attributed to withdrawal intervention.

**Conclusions:**

Our antimicrobial withdrawal intervention was a safe and effective addition to ASP activities to reduce inappropriate antimicrobial use and improve prescriber accountability.

## Introduction

Curbing inappropriate antimicrobial use is a major goal of hospital antimicrobial stewardship programs (ASP). An estimated 20–30 percent of antibiotics prescribed for hospital admissions could be safely discontinued, however, fewer than 10 percent are discontinued in practice.^1^ Several factors contribute to continuation of unnecessary antimicrobials in the inpatient setting. These include patient-level factors such as frailty and comorbidities^1^, prescriber-level factors such as diagnostic uncertainty^2^ and fear of adverse clinical outcomes^3^, as well as institution-level factors such as prescribing culture^4^ and hierarchy in clinical decision-making.^5^ Delayed prescribing initiatives, while successful in reducing inappropriate antibiotic use in the ambulatory setting^4^, have limited applicability in the fast-paced inpatient setting.

Consensus guidelines^6^ for implementing ASP stress the importance of persuasive or enforced prompting for interventions to be successful. Central to this process is persuasion theory^7^, which is based on using the target prescriber’s beliefs, values, and motives to change prescribing behavior. For example, leveraging prescribers’ motives to follow evidence-based recommendations and avoid negative consequences of noncompliance^8^ can be used to decrease inappropriate antibiotic prescribing behavior. Antibiotic stewards routinely utilize motivational interviewing, a persuasive, noncoercive method to achieve behavior change, when performing prospective audit and feedback (PAF).^8^ However, traditional PAF is limited by its non-compulsory nature, particularly for unrestricted formulary agents, which ultimately results in prolonged, unnecessary antimicrobial exposure.

ASP-driven antibiotic discontinuation targeting prolonged or duplicate therapy decreases excess antibiotic days and reduces burden on PAF.^9^ Herein, we aim to describe our first-year, single-center experience with a novel antimicrobial withdrawal policy to target inappropriately prescribed antimicrobials with the goals of reducing unnecessary antimicrobial exposure, as well as enhancing prescriber communication and accountability.

## Methods

### Setting, design, and participants

Lenox Hill Hospital (LHH) is a 450-bed tertiary academic medical center in New York City, NY, USA. The LHH ASP is co-led by an infectious diseases (ID) physician and ID pharmacist (hereafter referred to as ASP physician and ASP pharmacist, respectively) and uses a combination of formulary restriction with prior authorization and PAF, as per evidence-based consensus guidelines.^6^ We conducted a descriptive case series of adult inpatients admitted to LHH between December 21, 2021 and December 21, 2022 and included all patients on whom the antimicrobial withdrawal intervention described below was implemented. This study was not considered human subjects research by the Northwell Health Human Research Protection Program.

### Intervention

We determined appropriateness of antimicrobial therapy based on concordance of prescribing with previously developed evidence-based institutional guidelines (or Infectious Diseases Society of America guidelines where institutional guidelines were not available). We identified a subset of cases of inappropriate antimicrobial use historically challenging to address via traditional PAF—these included cases where antimicrobials were empirically prescribed and continued without confirmatory evidence of infection, prolonged antimicrobial therapy for clinically resolved confirmed infections, and prolonged postsurgical antibiotic prophylaxis. To address this subset, we developed a policy for withdrawal of inappropriately prescribed antimicrobials, which was vetted by hospital leadership, ASP Committee, and Pharmacy and Therapeutics Committee, and passed by the hospital’s Medical Board on December 14, 2021.

**Figure 1.**
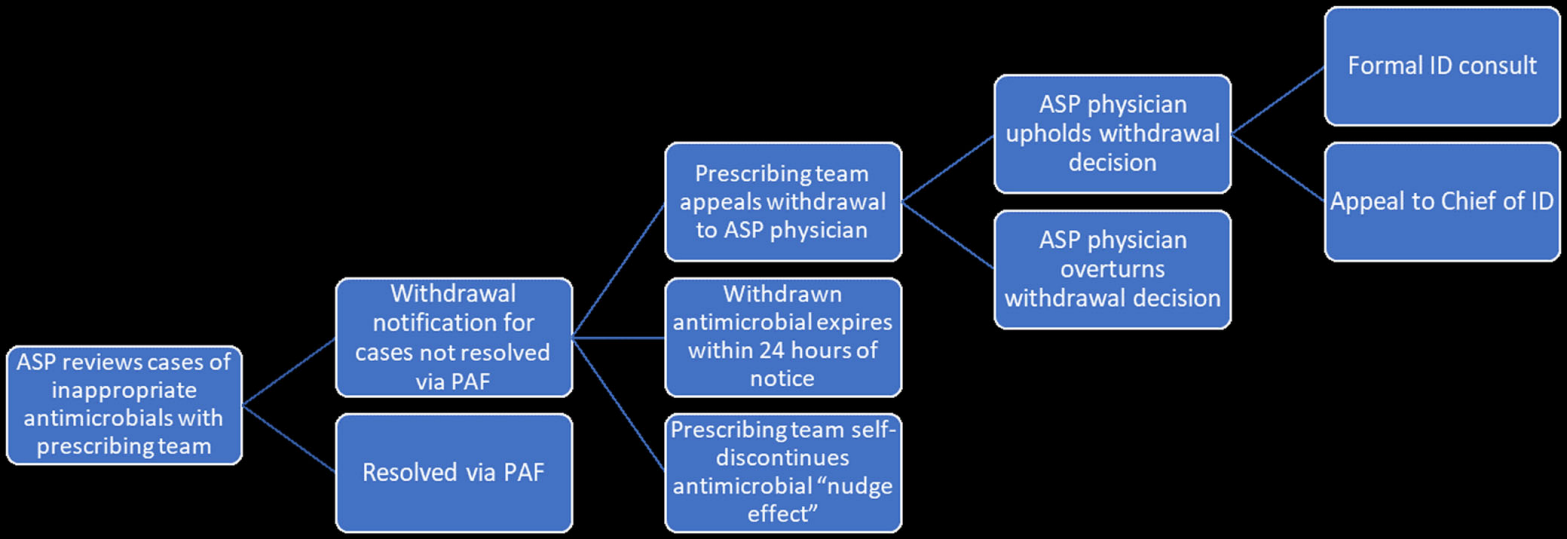
Flow Diagram of Antimicrobial Withdrawal Intervention. Abbreviations: ASP (antimicrobial stewardship program); PAF (prospective audit and feedback).

Cases of inappropriate antimicrobial use falling into the above categories were brought to the ASP physician by the ASP pharmacist during PAF rounds (occurring, on average, twice weekly, in hour-long sessions). Cases were selected for PAF rounds from a daily report of patients with active antimicrobial orders for six or more consecutive days (with the goal of focusing on prolonged prescriptions given time and personnel constraints); when time permitted, additional cases were reviewed from a report of active antimicrobial orders between three to five consecutive days or, on a case-by-case basis, at any point from therapy start date. The ASP physician reviewed and discussed each case with a member of the prescribing team. If the ASP physician determined use of the antimicrobial in question to be unjustified following discussion, a formal “withdrawal of anti-infective” notice was verbally given to the prescribing team and documented in the electronic medical record (EMR). Following this process, the primary team was allotted 24 hours to appeal the withdrawal decision to the ASP physician before the antimicrobial order in question expired. If the ASP physician determined use of the antimicrobial in question was still unjustified for cases appealed, the primary team was provided the opportunity to pursue a formal ID consultation or appeal again directly to the Chief of the Division of Infectious Diseases. Figure 1 presents a flow diagram of the withdrawal intervention. Key points of the withdrawal policy text can be found under supplemental materials.

### Process measures

We performed chart abstraction of the EMR to obtain data on the following prespecified process measures: count, date, and generic name of antimicrobials withdrawn; withdrawal category (no evidence of infection, prolonged duration of therapy or prophylaxis, or other); withdrawals by hospital service (surgical (a composite of cardiothoracic surgery, general surgery, surgical intensive care unit, vascular surgery, neurosurgery, orthopedics, plastic surgery) or medical (a composite of hospital medicine, cardiac care unit, medical intensive care unit, medical stepdown unit, cardiology)); time from antimicrobial start date to withdrawal intervention; days of therapy (DOT) saved (calculated as the difference between observed DOT with withdrawal and expected DOT without withdrawal (based on predefined treatment duration in orders or documentation or, if not defined, on clinical indication, with censoring at death or hospital discharge)); “nudge effect” defined as the prescribing team self-discontinuing withdrawn antimicrobial within 24 hours of withdrawal notice (a surrogate measure of prescriber concordance with ASP withdrawal decision); number of appeals to withdrawal; ordering of alternative antimicrobials following withdrawal.

### Outcome measures

We performed chart abstraction of the EMR to obtain data on the following prespecified outcome measures: incident infections, readmission, and in-hospital mortality up to 30 days following withdrawal.

### Statistical analysis

Baseline characteristics, process, and outcome measures were described using proportions/percentages, median/interquartile range (IQR), or mean/standard deviation as appropriate. A timeline of interventions by quarter was created using Microsoft Excel.

## Results

**Figure 2.**
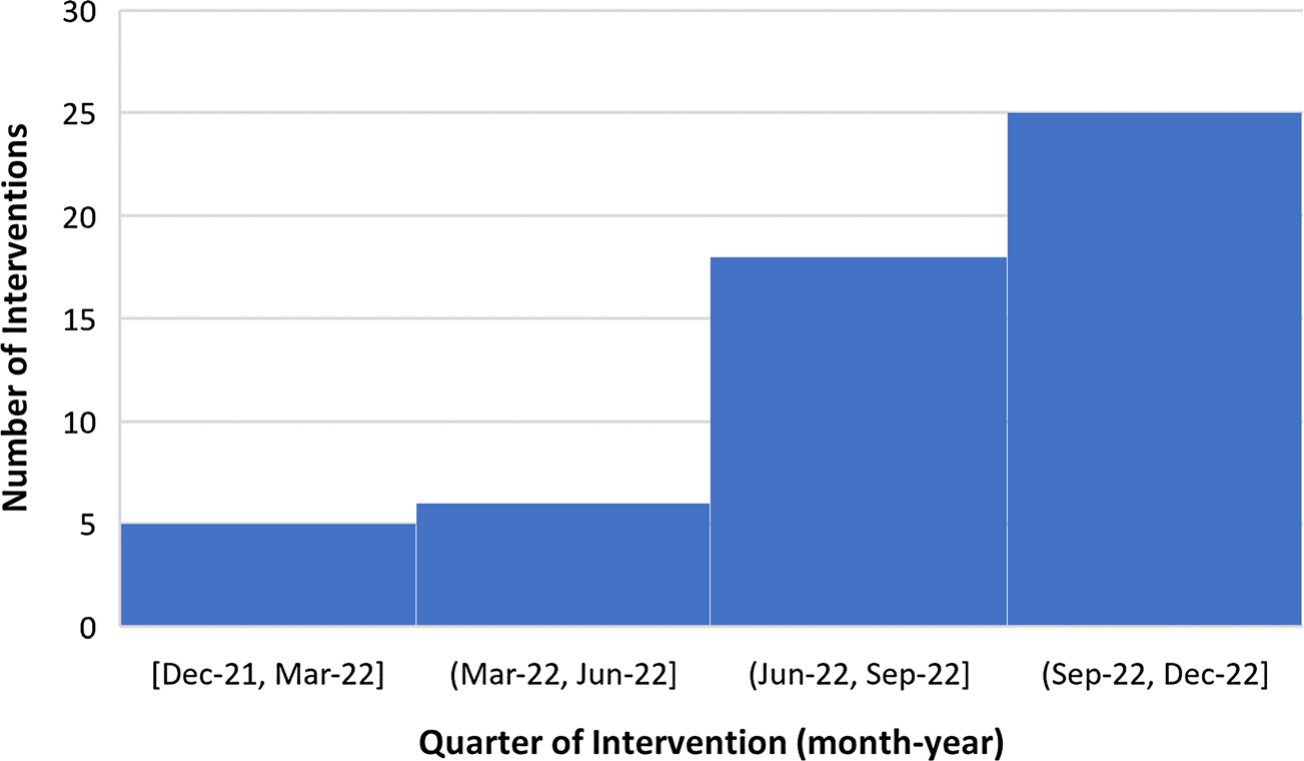
Antimicrobial Withdrawal Interventions by Quarter. Withdrawal interventions by quarter during the study period December 2021 to December 2022 are shown. Number of interventions is shown on the *y* axis.

During the study period from December 21, 2021 to December 21, 2022, of an estimated 278 cases reviewed during PAF rounds, there were a total of 54 antimicrobials withdrawn among 36 unique patients (on average, 4.5 withdrawals per month, 1.5 withdrawals per patient). A timeline of interventions is shown in Figure 2. Among the 36 patients, the mean age was 67.4 years (standard deviation 13.8 years) and 16/36 (44.4%) were female sex. Baseline characteristics, process measures, and outcome measures are summarized in Table 1.

**Table 1.** Baseline characteristics, process measures, and outcome measures

Measure	Value
Mean age (standard deviation) in years, n = 36 unique patients	67.4 (13.8)
Female sex (%), n = 36 unique patients	16 (44.4)
Antimicrobials withdrawn (%), n = 54	
Piperacillin-tazobactam	19 (35.2)
Vancomycin	8 (14.8)
Ceftriaxone	5 (9.3)
Cefazolin	5 (9.3)
Metronidazole	3 (5.6)
Meropenem	2 (3.7)
Other	12 (22.1)
Reason for withdrawal (%), n = 54	
Prolonged duration of therapy or prophylaxis	30 (55.6)
No evidence of infection	19 (35.2)
Other	5 (9.3)
Withdrawals by service (%), n = 54	
Surgical	41 (75.9)
Medical	13 (24.1)
Median time (interquartile range) in days from antimicrobial start date to withdrawal intervention, n = 54	6 (2–8)
Days of therapy (DOT) saved (DOT saved/100 patient-days)	236 (27.2)
Nudge effect (%), n = 54	13 (24.1)
Appeals to withdrawal (%), n = 54	17 (31.5)
Appealed to stewardship physician only	7 (13.0)
Appealed via ID consultation	9 (16.7)
Appealed to Chief of ID	1 (1.9)
Ordering of alternative antimicrobials after withdrawal (%), n = 54	11 (20.4)
New infections within 30 d of withdrawal (%), n = 36	4 (11.1)
Readmission within 30 d of withdrawal (%), n = 36	2 (5.6)
In-hospital death within 30 d of withdrawal (%), n = 36	6 (16.7)

Piperacillin-tazobactam was the most frequently withdrawn agent (19 (35.2%)), followed by vancomycin (8 (14.8%)), ceftriaxone (5 (9.3%)), cefazolin (5 (9.3%)), metronidazole (3 (5.6%)), meropenem (2 (3.7%)), and one each of daptomycin, clarithromycin, azithromycin, ciprofloxacin, levofloxacin, trimethoprim-sulfamethoxazole, nitrofurantoin, cephalexin, cefuroxime, amoxicillin-clavulanate, fluconazole, and caspofungin (22.1%).

The most common reasons for withdrawal were prolonged duration of therapy or prophylaxis (30 (55.6%)) and no evidence of infection (19 (35.2%)). Three agents (5.6%) were withdrawn because de-escalation of therapy was indicated, and two agents (3.7%) were withdrawn because potential harms of antimicrobial exposure were determined to outweigh potential benefits of continuation. Withdrawals occurred more frequently on surgical services (41 (75.9%)) compared to medical services (13 (24.1%)).

The median time from antimicrobial start date to withdrawal intervention was 6 days (IQR 2–8 days, range 0–22 days). An estimated 236 DOT (27.2 DOT per 100 patient days) were saved during the study period.

A nudge effect (defined in the methods section under process measures above) was seen in 13/54 (24.1%) of withdrawals. A total of 17/54 (31.5%) of withdrawals were appealed: 7/54 (13.0%) were appealed only to the ASP physician, with withdrawal decision upheld in 6/7 (85.7%) of cases; 9/54 (16.7%) were appealed via formal ID consultation, with withdrawal decision upheld in 7/9 (77.8%) of cases; only one case was appealed to the Chief of ID who upheld the withdrawal decision after reviewing the case with the appealing prescriber. Most appeals were based on primary team preference to extend empiric therapy or prolonged prophylaxis without objective clinical indication.

Ordering of alternative antimicrobials by the prescribing team following withdrawal was observed in 11/54 (20.4%) cases. The prescribing team ordered an oral antibiotic following withdrawal of an intravenous antibiotic in eight of these cases and switched to an alternative intravenous antibiotic in the remaining three cases. There were five other cases in which an alternative antimicrobial was recommended by ASP physician or ID consultant for appropriate escalation or de-escalation of therapy.

New infections were diagnosed in 4/36 (11.1%) of patients within 30 days of the index withdrawal. In the first case, vancomycin and piperacillin-tazobactam were restarted for possible hospital-acquired pneumonia one week after withdrawal of these agents, initially prescribed for soft tissue infection but with no evidence of soft tissue infection at that time—sputum cultures grew *Stenotrophomonas maltophilia* for which the patient was switched to trimethoprim-sulfamethoxazole. In the second case, cephalexin was empirically started for suspected surgical site infection four days following withdrawal of piperacillin-tazobactam prescribed with indication of urinary tract infection (UTI) due to no evidence of UTI at that time. In the third case, meropenem was started for *Klebsiella pneumoniae* bloodstream infection secondary to UTI one day after withdrawal of nitrofurantoin, which had been inadvertently prescribed as a home medication and was contraindicated due to renal impairment. In the fourth case, vancomycin was restarted for a new upper extremity thrombophlebitis four days following withdrawal for an uninfected groin wound. Incident infections were not definitively attributed to preceding withdrawal intervention in any of these cases.

Two patients were readmitted within 30 days of withdrawal intervention for reasons unrelated to the indication for antibiotic use during the prior admission. Six deaths occurred within 30 days of withdrawal intervention following removal of invasive life-prolonging measures in accordance with goals of care. None of the above readmissions and in-hospital deaths were attributed to preceding withdrawal intervention.

## Discussion

We present our first-year experience with a novel antimicrobial withdrawal intervention designed to reach high-hanging fruit in hospital antimicrobial stewardship—including cases of empiric antimicrobial use without evidence of underlying infection, prolonged antimicrobial therapy for confirmed, clinically resolved infections, and prolonged postsurgical antimicrobial prophylaxis. We demonstrate our policy to be an infrequent but useful adjunct to address cases historically challenging to approach via traditional PAF.

Piperacillin-tazobactam followed by vancomycin were the most frequently withdrawn agents, together comprising half of all interventions. Both agents are unrestricted on our hospital formulary and are the most prescribed antibiotics for suspected healthcare-associated infections at our institution. Broad-spectrum antibiotic use has been previously described as a risk factor for nonadherence to PAF—in one cohort^10^, piperacillin-tazobactam use was associated with 55% lower odds of adherence to PAF. Prolonged duration of therapy for confirmed, resolved infections, and prolonged postsurgical prophylaxis (ie, cefazolin prophylaxis for presence of surgical drain) were the most common reasons for withdrawal intervention. Prolonged duration of therapy has been previously used as a target for ASP-driven antibiotic discontinuation, resulting in significant decreases in mean total antibiotic days.^9^

The majority (∼76%) of withdrawal interventions occurred on surgical services. The disproportionate number of interventions is consistent with prior stewardship literature on suboptimal antibiotic prescribing among surgical services. A qualitative study^3^ of inpatient surgical teams identified antibiotic decision-making to be an uncoordinated, lower-priority task and described a general ambiguity in management of infections. In the same study, some surgeons described themselves as interventionists who acknowledged defensively prescribing antibiotics for fear of adverse postsurgical outcomes. An intervention^5^ designed to reduce inappropriately prolonged antibiotic use in surgical patients with source control also found the hierarchical nature of clinical decision-making within surgical teams to be a barrier to optimization of antibiotic therapy, with junior team members feeling powerless to influence more senior team members’ prescribing practices.

Our withdrawal policy was designed with the goals of enhancing prescriber communication and accountability over antibiotic prescribing. We found a so-called “nudge effect” following a quarter of interventions, where the prescribing team self-discontinued the antimicrobial in question and interpreted this as a surrogate for prescriber concordance with ASP withdrawal decision; in fact, in a few instances, frontline prescribers were grateful for ASP intervention in providing leverage against more senior team members to discontinue unnecessary therapy. Our policy allowed the prescribing team a 24-hour window to appeal the withdrawal decision to the ASP physician. The appeal process was intended to create a flipped dynamic, where the prescribing team needed to justify continuation of antibiotic therapy to ASP (in contrast to ASP justifying discontinuation of therapy to prescribing team); we believe this process enhanced intra-team communication (ie, between junior frontline prescribers and more senior team members) and improved prescriber answerability and transparency.

In response to the minority of cases of reordering of the withdrawn agent (or ordering of an alternative antimicrobial), a clause was added to the withdrawal policy after the end of the study period to allow the ASP to immediately discontinue inappropriately re/ordered agents within 48 hours of intervention. We found our withdrawal intervention reduced burden on PAF by not revisiting cases where recommendations were previously not accepted, consistent with a previously published ASP-driven antibiotic discontinuation intervention.^9^ However, we found this reduction was partially offset by increased burden on the ASP physician needing to address appeals and re/ordering of antimicrobials. We found limited burden on the busy ID consult service from appeals to withdrawal and reassuringly high concordance between withdrawal decision and ID consultant recommendations.

We found reassuring safety of our withdrawal intervention with respect to incident infections, readmission, and in-hospital mortality within 30 days of withdrawal. In one case, nitrofurantoin, while inappropriately prescribed, may have been suppressing growth of *Klebsiella* in a patient who developed bacteremia following its withdrawal; while nitrofurantoin susceptibility testing was not performed on this blood isolate, nearly half of all *Klebsiella* cultured from urine are resistant to this agent at our institution. No readmissions or deaths were attributed to preceding withdrawal intervention. An important caveat, however, is that we could not verify if patients were readmitted to another hospital or died following hospital discharge. A study^11^ of 794 ASP interventions over a five-year period assessing the safety of ASP discontinuation of empiric antibiotics within 24 hours of prescribing in patients with no evidence of bacterial infection found significantly decreased DOT and length of stay and no difference in infection-related readmission or all-cause 30-day mortality.

Our intervention had some strengths: flexibility to intervene at any point during the course of prescribed therapy; discretion by ASP physician to intervene for multiple categories of inappropriate antimicrobial use; broad range with applicability to both formulary-restricted and unrestricted antimicrobials; wide reach across all inpatient services; enforceability via hospital policy and support from hospital leadership; appeal process to improve prescriber accountability over antimicrobial prescribing.

Our intervention was not without limitations. As this was a descriptive case series of an intervention without a comparison group, causal inferences regarding withdrawal outcomes cannot be drawn. Our experience may not be applicable to other centers. Our policy was introduced years after the ASP developed rapport and credibility with prescribers and leadership across the hospital. We emphasize this policy should be used as an infrequent adjunct and not as a substitute to traditional ASP activities including PAF, formulary restriction with prior authorization, and antimicrobial prescribing guidance development (eg, for postsurgical antibiotic treatment or prophylaxis). Judicious use is especially important when weighing respect for prescriber autonomy with concern for safety and quality of antimicrobial prescribing as an overly restrictive approach can lead to loss of trust between prescribers and stewards;^12^ this balancing act is not new for stewardship programs.

We present our early experience with an innovative antimicrobial withdrawal intervention that effectively targeted inappropriate inpatient antimicrobial use and improved prescriber accountability over antimicrobial prescribing. Our policy was found to be a safe and useful addition to traditional ASP activities. Continued follow up will be needed to gauge sustainability and long-term impact.

## Supporting information

Khan et al. supplementary material Khan et al. supplementary material

## Supplementary material

For supplementary material accompanying this paper visit http://doi.org/10.1017/ash.2024.48. click here to view supplementary material
