# Supplementary material for: Reaching high-hanging fruit in antimicrobial stewardship: a hospital-based intervention to withdraw inappropriately prescribed antimicrobials
Source: Antimicrob Steward Healthc Epidemiol. 2024 Apr 16;4(1):e42. doi: 10.1017/ash.2024.48 (PMC11019577; doi:10.1017/ash.2024.48)
Supplement: Khan et al. supplementary material [file S2732494X24000482sup001.docx]

Key Points of the Withdrawal Policy Text

- If, at the discretion of the Antimicrobial Stewardship Physician, prescribed antimicrobials are determined to be inappropriate, the Antimicrobial Stewardship team will contact the primary team to verbally address concerns and recommend cessation of antimicrobial in question and a 24-hour expiration notice will be placed in the computerized provider order entry (CPOE) by the Antimicrobial Stewardship pharmacist.
- The primary team will have 24 hours (or in the case of after hours, holiday or weekend, the next business day) to appeal the decision to the Antimicrobial Stewardship Physician.
- If the Antimicrobial Stewardship Physician determines that use of the antimicrobial in question is still unjustified, a formal ID consultation will be recommended.
- If the primary team declines recommendation for formal ID consult, then the next step will be to appeal (on the same day) to the Chief of ID.
